# Rhizospheric microbiomes differ between dormant and active *Potaninia mongolica* in the Gobi desert of Mongolia

**DOI:** 10.1038/s41598-025-22696-7

**Published:** 2025-11-06

**Authors:** Batdelger Erdenetsetseg, Kazuharu Arakawa, Josephine Galipon, Sainbileg Undrakhbold, Shinji Fukuda, Bazartseren Boldgiv

**Affiliations:** 1 Department of Biology, National University of Mongolia, Ulaanbaatar, Mongolia; 2 Institute for Advanced Biosciences, Keio University, Tsuruoka, Japan; 3 Graduate School of Media and Governance, Keio University, Fujisawa, Japan; 4 Faculty of Environment and Information Studies, Keio University, Fujisawa, Japan; 5 Graduate School of Science and Engineering, Yamagata University, Yonezawa, Japan; 6 Gut Environmental Design Group, Kanagawa Institute of Industrial Science and Technology, Kawasaki, Japan; 7 Innovative Microbiome Therapy Research Center, Graduate School of Medicine, Juntendo University, Tokyo, Japan; 8 Transborder Medical Research Center, University of Tsukuba, Tsukuba, Japan

**Keywords:** Rhizosphere, *Potaninia mongolica*, Bacteria, Fungi, Microbiome, Gobi desert, Microbiome, Microbial ecology

## Abstract

Rhizospheric microbiomes differ between active and dormant plants due to changes in root activity and exudate production, especially under environmental stress. In arid regions, native plants such as *Potaninia mongolica* Maxim enter dormancy to survive harsh conditions. However, rhizospheric microbial and chemical differences between active and dormant states of plants remain poorly described. This study investigated rhizospheric microbial communities and soil chemical changes in the case of active and dormant *P.mongolica* plants. Rhizospheric soil samples were collected, and soil texture and chemical variables were analyzed. High-throughput sequencing targeting the 16S rRNA and ITS regions was conducted to profile bacterial and fungal communities, respectively. Results showed that the dominant fungal phyla were Ascomycota and Basidiomycota, while Proteobacteria and Actinobacteria were the dominant bacterial phyla in both plant states. Although bacterial diversity did not differ significantly between active and dormant plants (p > 0.05, Welch’s t-test), fungal diversity was significantly different. Among soil chemical variables, total nitrogen was notably elevated in the rhizosphere of dormant plants (mean = 7.93; SD = 5.91). These findings reveal differences in fungal community structure and nitrogen levels in the rhizosphere between active and dormant plant states. Understanding these interactions contributes to our knowledge of desert plant microbiome dynamics and may inform the use of microbial indicators or amendments to support vegetation restoration in arid environments.

**Supplementary Information:**

The online version contains supplementary material available at 10.1038/s41598-025-22696-7.

## Introduction

Plants, as the foundation of terrestrial ecosystems, anchor themselves in soil via their roots, contribute to food webs, and help prevent soil erosion. Their survival in harsh environments is facilitated by a range of adaptive strategies, including interactions with soil microorganisms in the rhizosphere the narrow zone surrounding roots^1^. These microbial communities, known collectively as the rhizobiome, play essential roles in plant nutrient uptake, immunity, and stress resistance. However, not all soil microbes are beneficial, host plants influence the composition of the rhizobiome by selectively recruiting taxa that support their health and defense^2^. Understanding how plants shape and benefit from their microbiomes remains a key focus of current research.

Soil fungi and bacteria both play critical but distinct roles in supporting plant growth and resilience. Fungi are known for their ability to decompose complex organic materials and are highly responsive to changes in root exudates and host-derived carbon^3^^,^ while bacteria often display broader metabolic flexibility, enabling persistence under a wider range of conditions. In arid ecosystems, rhizospheric microbes are particularly important for plant adaptation to extreme stress^4^. Fungal symbionts such as arbuscular mycorrhizal fungi (AMF) respond dynamically to plant activity^5^^,^ and recent studies show that plant diversity is positively correlated with fungal diversity, especially AMF and saprotrophic fungi, mediated by soil nutrient status and texture^6^. These relationships are further influenced by the physical and chemical properties of the soil, which interact with microbial activity and distribution to shape ecosystem processes.

The Gobi Desert is Asia’s largest and the world’s sixth largest desert hosts a unique and fragile ecosystem across southern Mongolia and northern China^7^. Among its rare endemic flora is *Potaninia mongolica*, the sole species of its genus in the Rosaceae family^8^. This desert shrub is adapted to extreme aridity and is listed as endangered in the Red List of Mongolia^9,10^. It grows in gravelly and sandy soils in desert steppe regions and exhibits remarkable dormancy behavior, allowing it to remain inactive during unfavorable conditions and regenerate after rainfall^11^. Though propagation is possible via seed, fissuration, or root cuttings, natural regeneration is limited, contributing to its endangered status (Quanlin et al.^91^).

One distinctive feature of *P. mongolica* is that both dormant and active individuals can be found in close proximity within the same habitat, suggesting that localized environmental or biological factors influence plant activity. However, the specific role of rhizospheric microbial communities in this phenomenon remains unclear. Given that rhizospheric fungi and bacteria are involved in nutrient cycling, stress tolerance, and plant signaling, they may be central to *P. mongolica*’s adaptation and persistence in desert conditions. Rare plants hold high conservation significance due to its ecological uniqueness and limited distribution. However, its long-term survival may depend not only on habitat protection but also on a deeper understanding of its belowground microbial associations. The rhizospheric microbiome comprising fungi and bacteria involved in nutrient cycling, stress tolerance, and root health may play a pivotal role *in P. mongolica*’s ability to persist under harsh desert conditions. By exploring the structure and function of its microbiome, particularly in relation to plant activity and dormancy, this study provides insights that could inform strategies for ecological restoration, ex-situ propagation, and climate-resilient conservation management of this endangered species.

This study investigates the composition and diversity of rhizospheric microbial communities (both bacterial and fungal) associated with dormant and active *P. mongolica* individuals in the Gobi Desert. We used amplicon-targeted metagenomics^12^ to profile the microbiome and compare it against background soil communities. Our specific objectives were to: (i) compare the distribution of rhizospheric bacteria and fungi between dormant and active plant states, and (ii) identify microbial taxa potentially involved in supporting plant dormancy or reactivation, alongside associated soil properties. Insights gained from this study may inform conservation strategies, ex-situ propagation, and ecosystem restoration efforts for this ecologically important and endangered desert shrub.

## Study area

**Fig. 1:**
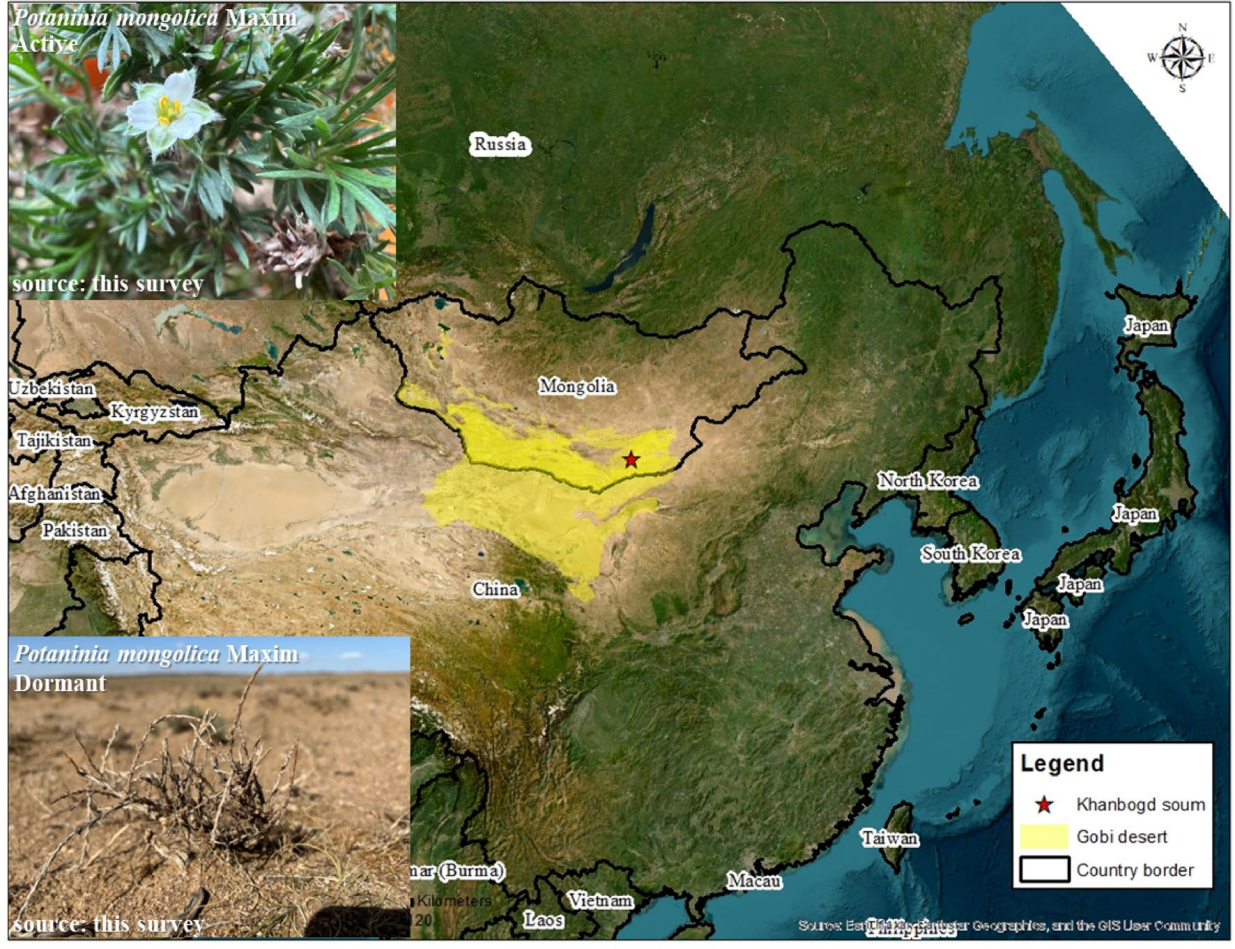
Location of the study area. The survey area is marked in red, with the Gobi Desert region indicated in yellow (The map was created specifically for this survey by M. Uuganbayar, using the Ecosystem Type ^94^ data from the WWF Mongolia open-source database). The map was created using ArcGIS 10.4 with satellite imagery as the base layer. The top-left photo shows an active plant, while the bottom-left photo shows a dormant plant.

The research was carried out in the area of Khanbogd soum (43.203028N 107.178528E) (a soum is an administrative unit in Mongolia and can be understood as a district) in the southern Gobi Desert of Mongolia (Fig. 1). The soum occupies 15,150 km^2^ and belongs to the phytogeographic region of Alashaa Gobi, Dornogobi steppe and Gobi-Altai mountain dessert steppe. About 180 species of vascular plants are found within the soum. Vegetation in this region consists primarily of drought-tolerant edificator and sub-shrub species that characterize the typical desert physiognomy. Dominant species include *Eurotia ceratoides*, *Brachanthemum gobicum*, *Potaninia mongolica*, and *Sympegma regelii*, along with desert-adapted subshrubs such as *Anabasis brevifolia* and *Salsola passerina*^13,14^. The Gobi brown soil and desert brown-gray soil are predominant in the area. One of the largest alkali granite plutons in the world is located in this soum. It is positioned at the heart of the Late Paleozoic Syncline^15^. The area is inhabited by such rare wildlife species such as the Asiatic wild ass and goitered gazelle. In terms of climate, Khanbogd soum has an extreme seasonal climate, with temperatures reaching up to + 40 degrees Celsius in summer and −15–25 degrees Celsius in winter. Average annual precipitation is 95.3 mm^16^. The soum is the warmest place in Mongolia. The Galba Gobi, a part of this soum, is a favored habitat for domestic Bactrian camels, and Khanbogd soum has the highest number of camels in Mongolia. There are also abundant resources of copper, molybdenum, gold and coal in the area, and there is a large copper mine, Oyu Tolgoi, that operates in the soum.

## Methods

### Sampling and DNA extraction

**Fig. 2:**
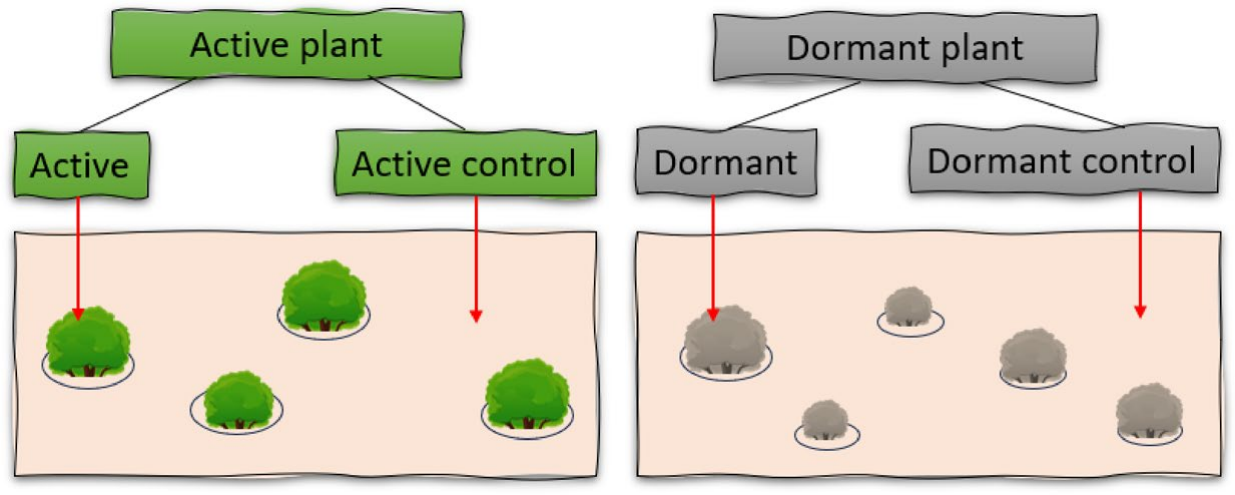
Representation of the rhizosphere sampling process from dormant and active plant with their respective control.

Soil samples were taken from each active and dormant plant to compare the rhizospheric bacterial and fungal communities with their respective controls. Control samples were collected from the open area at 4 replicates, away from the host plant and other plants (Fig. 2). Root-associated samples from both active and dormant plants were collected, with eight biological replicates. Each habitat included at least three biological replicates, consistent with standard practices in microbiome studies conducted in arid environments (Fig. 2)^17^. Dormant and green covered individuals of *P. mongolica* were selected and DNA was extracted from the soil around the roots using the Extrap Soil DNA Kit Plus V2 (Funakoshi, Japan), according to the manufacturer’s instructions. The plant was formally identified by Prof. B. Oyuntsetseg and Prof. R. Tungalag, botanists at the National University of Mongolia with expertise in native flora. A reference voucher specimen, collected and identified by Prof. B. Oyuntsetseg, has been deposited in the publicly available herbarium of the National University of Mongolia under the deposition number UBU0012713.

### PCR amplification of 16S V1-V2 and ITS regions

Tks Gflex™ DNA Polymerase (Takara Bio, Inc., Japan) was used for PCR on the extracted DNA. Oligonucleotide purification cartridge (OPC)-grade primers were ordered from Eurofins Genomics (Japan), and their sequences are as follows: 16S V1-V2 region: [forward primer 27Fmod: 5′-ACA CTC TTT CCC TAC ACG ACG CTC TTC CGA TCT AGR GTT TGA TYM TGG CTC AG-3’] and [reverse primer: 5′-GTG ACT GGA GTT CAG ACG TGT GCT CTT CCG ATC TTG CTG CCT CCC GTA GGA GT-3’]^18^, ITS region: [forward primer u1: 5′-ACA CTC TTT CCC TAC ACG ACG CTC TTC CGA TCT GGA AGK ARA AGT CGT AAC AAG G-3] [reverse primer 338R: 5’-GTG ACT GGA GTT CAG ACG TGT GCT CTT CCG ATC TGC GTT CAA AGA YTC GAT GRT TC-3’]^19^. After confirming successful amplification without overamplification by agarose electrophoresis, the sequencing libraries for Illumina as previously published^20^. Briefly, after purifying the DNA with AMPure XP beads (Beckman Coulter, USA), the maximum amount of DNA in each sample was normalized to 4 nM, and 8 additional cycles of amplification were used to attach the Illumina barcodes. Parallel amplicon sequencing was performed on final amplicons normalized to 6 pM spiked with a 10% Phix Control v3 (Illumina, USA), using the Illumina MiSeq Reagent Kit v3 in paired-end 300 bp sequencing mode at the Institute for Advanced Biosciences, Keio University.

### Soil chemical analysis

The soil samples were taken from a depth of 10–15 cm by removing the topsoil. Hand shovels were sterilized with 99% ethanol before collecting each sample. The active and dormant plants are in the same environment in terms of soil structure and geological structure and are located on a uniform, flat surface. Soil samples were analyzed for twelve soil characteristics (nitrate nitrogen, pH, phosphate, total dissolved solids, salt, humus, absorbed Ca + Mg, absorbed Ca, absorbed Mg, potassium oxide, soil electrical conductivity, sulfate) and soil texture at the Soil Research Laboratory of the National University of Mongolia. The soil texture analysis was included to confirm that soil structure did not confound the observed differences in rhizospheric microbial communities between active and dormant plants. Soil samples were prepared and analyzed to determine key physicochemical parameters using the following methods: Soil pH was measured using a Mettler Toledo Seven Compact benchtop pH meter in a 1:5 (w/w) soil-to-water suspension. Humus content was quantified using I.V. Tyurin’s method, based on the oxidation–reduction principle in which organic matter is oxidized by potassium dichromate in sulfuric acid medium and subsequently titrated. This classical approach remains widely applied in Eastern European and Asian soil laboratories for characterizing soil organic matter fractions^21^. Nitrate nitrogen was extracted with a 1:5 water solution and quantified using ion chromatography. Phosphate was measured using B.P. Machigin’s method and a spectrophotometer. Potassium oxide was determined by Machigin’s flame photometric method, in which soil-extractable potassium is excited in a flame and its emission intensity is compared against standards. Flame photometry remains a reliable technique for available potassium estimation in agronomic and ecological studies^22^. Absorbed Ca + Mg were quantified by complexometric titration using Trilon-B as the titrant. This method allows simultaneous determination of bivalent cations and is widely applied in soil fertility assessments^23^. Electrical conductivity and total dissolved solids were measured from a 1:5 (w/w) soil–water extract.

### Bioinformatic processing and statistical analysis

Qiime2 ^93^.2 software was used to perform the bioinformatic and biostatistical analyses^24^. Default settings were used unless otherwise specified. The sequences of rhizospheric microbiomes of dormant and active plants were trimmed with left-forward 5, left-reverse 5,trunc-length-forward 290 and trunc-length-reverse 240 using DADA2 database. Our sampling depth was 10,000. Alpha diversity was then calculated using the Chao index. In the case of analysis using the 16S gene, the Silva 132 database was used. After downloading and extracting the entire Silva database, only the V1 and V2 regions were selected to prepare the database. For fungi, sh_refs_qiime_ver8_99_04.02.2020 database was used. For classification and taxonomy analysis, a combination of sequencing database and taxonomy database was used. Permutational multivariate analysis of variance (PERMANOVA), a non-parametric permutation test in multivariate statistics, compares groups of objects by testing the hypothesis that the centroids and dispersion of groups in the measured space are equivalent across all groups^25^. PERMANOVA was used to evaluate differences in microbial community structure among sample categories, with analyses performed in QIIME2. Indicator Species Analysis (De Cáceres and Legendre, 2009) was conducted to define the indicator fungal species responsible for the differences between the active and dormant plant soil around the root with respective control using *indispecies* package in RStudio 2024^92^.09.0 + 375. The correlation between the distribution of indicator species and soil factors was evaluated using Kendall’s τ test in JMP Pro 16 software (Kendall, 1938). Welch’s t-test was used to compare differences between the rhizospheres of active and dormant plants and their respective controls with JMP Pro 16 software**.** Jaccard’s index of similarity measures the similarity between two sets by comparing the number of elements they have in common^26^. The index ranges from 0 to 1. A range closer to 1 means more similarity in the two sets of data. The Jaccard index was calculated using the EstimateS _910_Windows software. To account for variations in sequencing depth across samples, rarefaction was performed prior to diversity analysis using RStudio 2024.09.0 + 375. For bacterial 16S rRNA gene data, sequences were rarefied to 10,000 reads per sample, while fungal ITS sequences were rarefied to 5,000 reads per sample. These thresholds were chosen based on rarefaction curve assessments to retain the majority of samples while minimizing loss of diversity information. Alpha diversity was quantified using the Chao1 index, a nonparametric estimator of species richness that incorporates rare taxa and thus provides a more accurate richness estimate^27^, while the Shannon index accounts for both richness and evenness, offering insight into community diversity structure. To evaluate differences in microbial community composition across samples, Bray–Curtis dissimilarity was used as the primary beta diversity metric. Bray–Curtis is particularly effective for detecting compositional differences between groups^28^.

## Results

### Soil chemicals

**Fig. 3:**
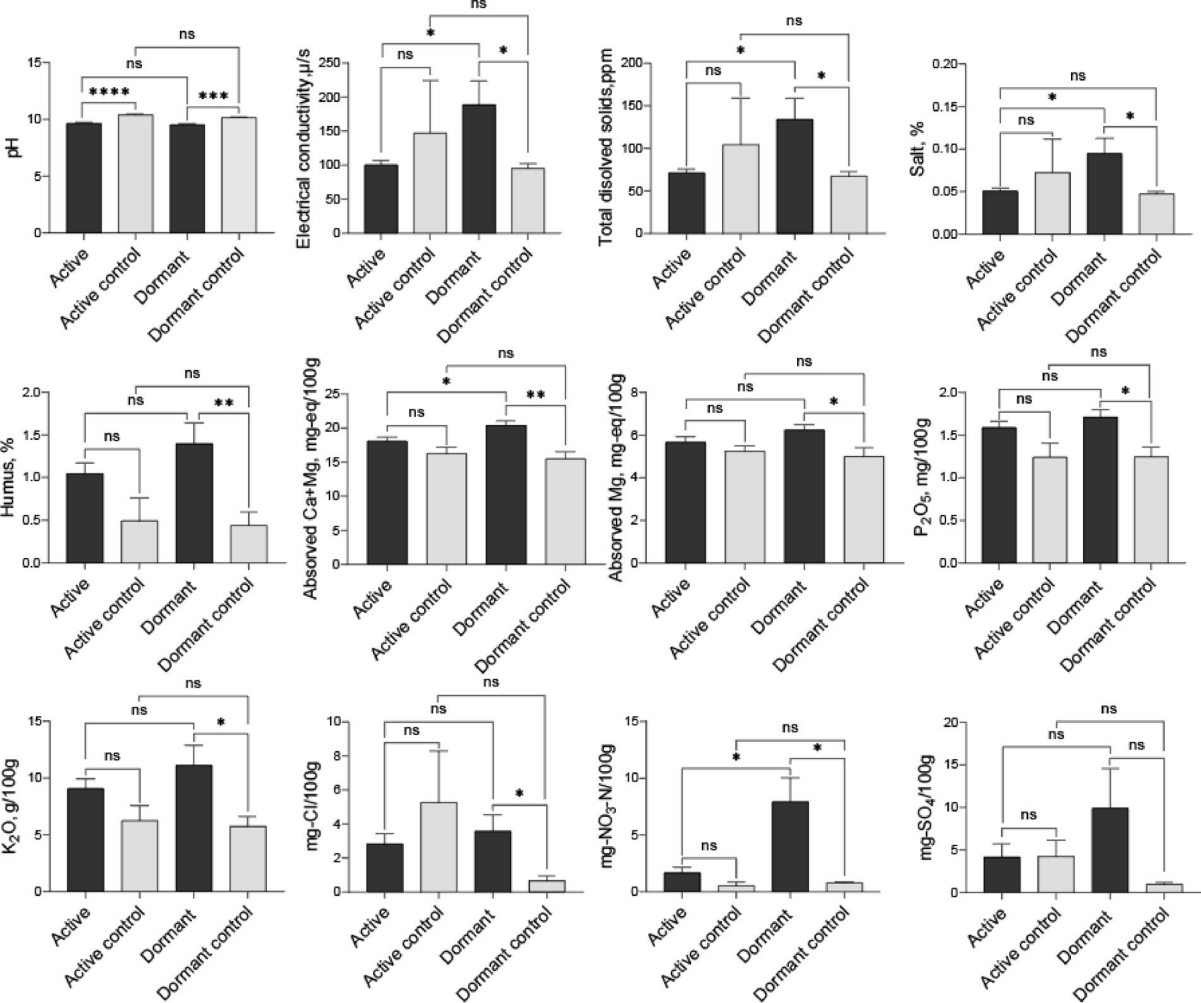
Result on the chemical analysis of soils collected from the rhizosphere of active and dormant plants. Welch’s t-test was used to compare differences between the states and their respective controls**.** Significant differences are indicated by the following significance codes: ‘****’ 0.0001, ‘***’ 0.001, ‘**’ 0.01, ‘*’ 0.05, ‘ns’ non-significant.

The soil pH around the root of active and dormant *P. mongolica* plants differed from their respective control, but did not differ each other. Conductivity, total disolved solids and salt were statistically different between active and dormant plant rhizospheres. For humus, there was no difference between dormant and active plants, but there was a difference between dormant and its control. Absorbed Ca + Mg mg-eq/100 g and absorbed Ca mg-eq/100 g were different between active and dormant. There is no difference in absorbed Mg mg-eq/100 g, ${\text{P}}_{2}$ ${\text{O}}_{5}$ mg/100 g, ${\text{K}}_{2}$ O mg/100 g and Cl mg/100 g in the soil around the root of dormant and active plants. In terms of ${\text{NO}}_{3}$-N mg/100 g, it was more abundant in the soil around the roots of the dormant and is much higher than that in the active. In the active plants, ${\text{SO}}_{4}$ mg/100 g did not differ between all treatments. The active control and dormant control were not significantly different in all soil chemicals (Fig. 3).

**Fig. 4:**
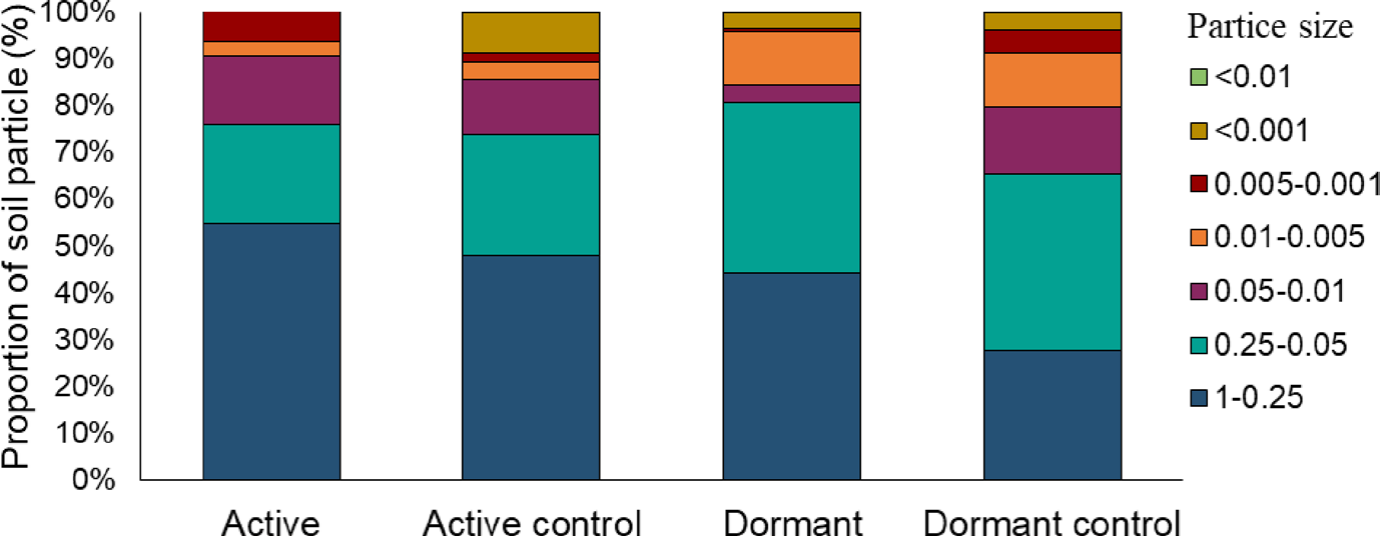
Particle size distribution as a percentage of the total particle volume. The distribution represents the proportion of soil particles within the sample, categorized by size fractions. Each segment indicates the percentage of particles within specific size ranges, providing insight into the texture and composition of the soil sample.

The proportion of soil particles in the 0.05–0.01 and 0.005–0.001 of the soil around the roots of dormant plants is relatively lower than in the others. However, Fig. 4 shows that the soil mechanical properties of all samples were similar. The soil for active, active control and dormant plants is loamy sand, while the soil for dormant plants is light loam.

### Bacterial identification (16S)

**Fig. 5:**
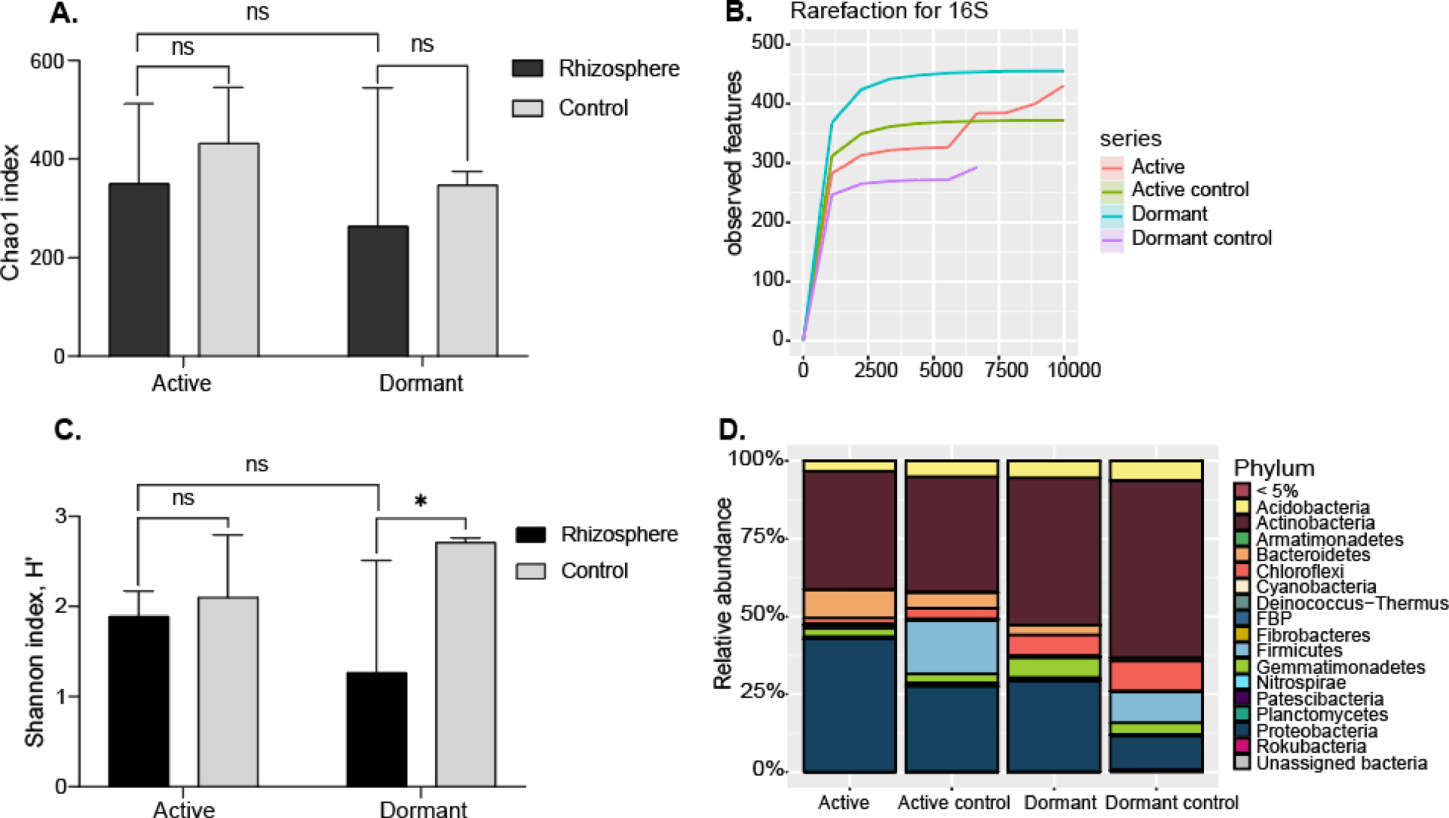
(**A**). Chao index for bacterial diversity in the rhizosphere of *Potaninia mongolica* during active and dormant states, compared to their respective controls. The x-axis represents plant states (active, dormant) and their respective controls, while the y-axis shows the Chao index values. Each bar represents the mean Chao index across replicates, with error bars indicating standard deviations. (**B**). Rarefaction curve analysis showing the depth of 16S sequencing of around root soil of active and dormant *Potaninia mongolica*. The x-axis represents the observed features, and the y-axis represents the the sequencing depth. (**C**). Shannon index for bacterial diversity in the rhizosphere of *P. mongolica* during active and dormant states, compared to their respective controls. The x-axis shows plant states, and the y-axis represents Shannon index values. Error bars indicate standard deviations. (**D**). Relative abundance of bacteria at the phylum level. The x-axis represents bacterial phyla, and the y-axis indicates the relative abundance as a percentage of the total bacterial community.

There were no significant differences in bacterial diversity in the soil around the roots of active and dormant plants (Fig. 5a). The rarefaction curves (Fig. 5b) tend to approach the saturation line, indicating that the obtained sequences were sufficient. The Shannon index revealed no significant difference in bacterial diversity between the rhizosphere of active plants and their respective controls, whereas the rhizosphere of dormant plants showed a statistically significant difference in diversity compared to its control (Fig. 5c).The relative abundances of bacteria at the phylum level in soil around the roots are presented in Fig. 5d (Supplementary data 1). According to the results, the dominant bacteria were Actinobacteria and Proteobacteria.

The similarity index of bacterial communities of active and active control plants was 79.8 percent, while the similarity index of bacterial communities of dormant and dormant control plants was 98.6. The similarity between active and dormant plants is 77.4 percent. The Bray–Curtis index was 59.3% between the rhizospheres of active and dormant plants, 64.1% between active plants and their respective controls, and 58.2% between dormant plants and their respective controls (Table 1).

**Table 1:** Jaccard index of similarity between the active and dormant phases in bacterial community.

	**Jaccard index**	**Bray–Curtis index**	**p-value (t-test**
active vs active control	0.798	0.641	ns
active vs dormant	0.774	0.593	ns
active vs dormant control	0.913	0.404	ns
active control vs dormant	0.992	0.635	ns
active control vs dormant control	0.994	0.551	ns
dormant vs dormant control	0.986	0.582	ns

Six indicator species were identified by IndVal in the active plant rhizospheric bacteria, three in the active control, one in the dormant state, and three in the dormant control. Three of the active plant indicator species belong to the Actinobacteria, two to the Proteobacteria, and one to the Bacteroidetes phylum. Two indicator species of the active control belong to the Firmicutes, and one to the Actinobacteria. Dormant plants indicator species belongs to the Actinobacteria phylum. Dormant control plants indicator rhizospheric bacterial species include one from the Proteobacteria, one from the Nitrospira, and the remaining two from the Chloroflexi (Table 2).

**Table 2:** List of bacterial indicator species with their IndVal percentage and *p*-value. Significance codes: ‘***’ 0.001, ‘**’ 0.01, ‘*’ 0.05.

States	Species	IndVal	*p*-value
Active	*Lechevalieria aerocolonigenes*	0.791	0.003 **
	*Kineococcus-like bacterium AS3382*	0.791	0.008**
	*Chitinophagaceae bacterium IBVUCB1*	0.791	0.005**
	*Belnapia rosea*	0.713	0.026*
	*Microvirga subterranea*	0.709	0.042*
	*Microbacterium yannicii PS01*	0.672	0.047*
Active control	*Bacillus polygoni*	0.723	0.028*
	*Bacillus foraminis*	0.713	0.047*
	*Blastococcus saxobsidens*	0.683	0.001***
Dormant	*Sporichthya polymorpha DSM 43,042*	0.707	0.028*
Dormant control	*Ramlibacter tataouinensis TTB310*	0.866	0.005**
	*Nitrospirae bacterium 13_2_20CM_2_62_8*	0.799	0.009**
	*Thermobaculum terrenum ATCC BAA-798*	0.707	0.044*

**Fig. 6:**
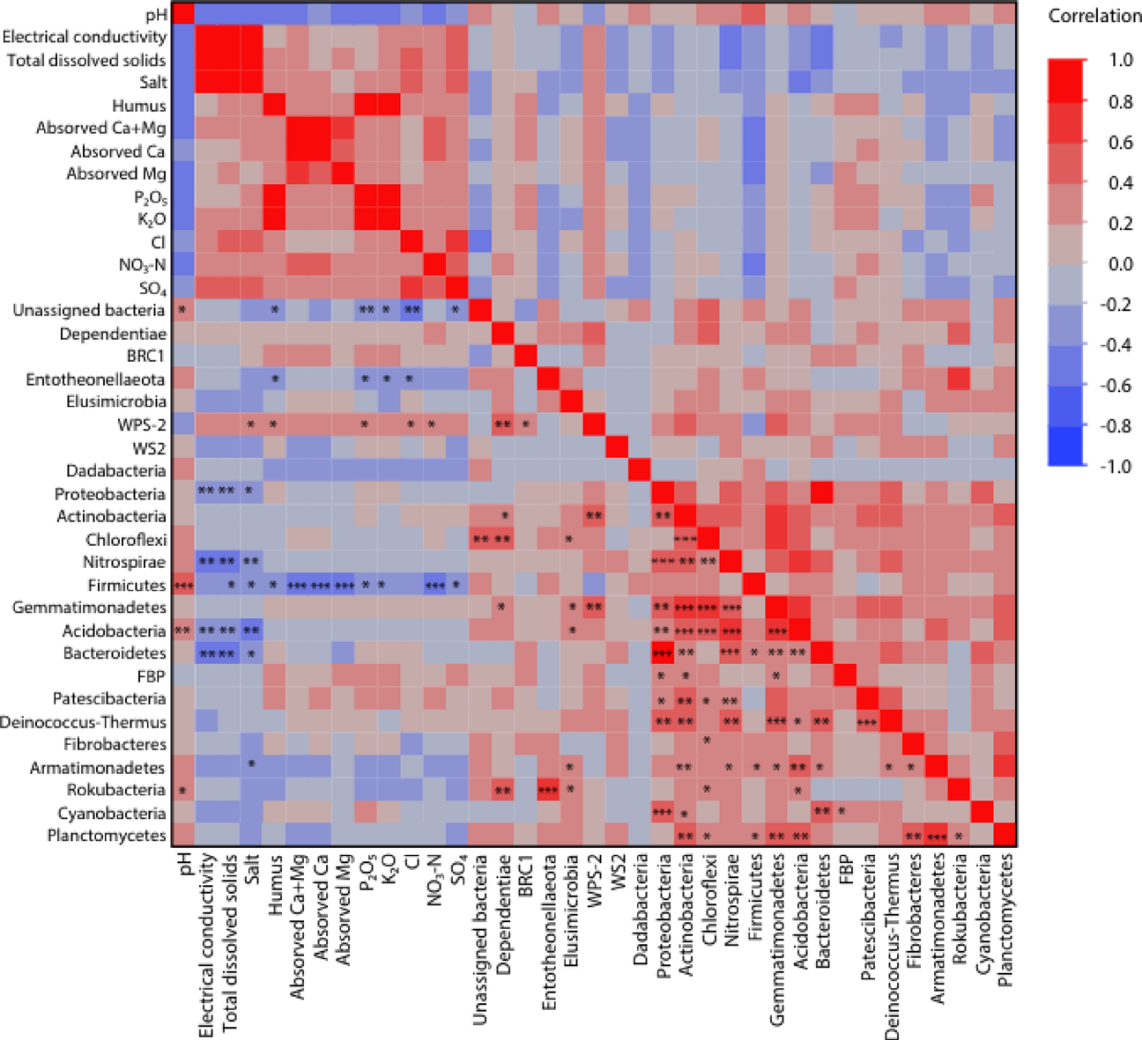
Heatmap of Kendall’s correlation coefficient, presenting associations of indicator bacterial abundance with each other and with soil chemicals. The heatmap illustrates the strength and direction of correlations, where colors represent the magnitude of Kendall’s coefficient, with warmer colors indicating positive correlations and cooler colors indicating negative correlations.

The correlation results showed a significant negative correlation between abundance of Entotheonellaeota and K₂O₅ (Kendall’s τ = −0.3595; *p* = 0.0454) (Fig. 6). Abundance of WPS-2 bacterial phylum showed a significant positive correlation with salt, humus, P₂O₅, Cl, and NO₃-N (Kendall’s τ = 0.3544, 0.3330, 0.3620, 0.3760, 0.3879; *p* = 0.0493, 0.0485, 0.0317, 0.0264, 0.0219). Abundance of BRC1 and WS2 bacterial phylum did not exhibit a significant correlation with soil chemicals. Abundance of FBP bacterial phylum was positively correlated with abundance of other phylum, such as Proteobacteria, Actinobacteria, Gemmatimonadetes (Kendall’s τ = 03,785, 0.36, 0.3316; *p* = 0.0214, 0.0293, 0.0458). Abundance of Proteobacteria showed a negative correlation with electrical conductivity and total dissolved solids (Kendall’s τ = −0.3534, −0.3461; *p* = 0.0160, 0.0183). Abundance of Nitrospirae were significantly negatively correlated with electrical conductivity, total dissolved solids, and salt (Kendall’s τ = −0.4520, −0.4446, −0.3820; *p* = 0.0023, 0.0028, 0.0164). Abundance of Firmicutes were positively correlated with pH (Kendall’s τ = 0.5275; *p* = 0.0005) and negatively correlated with salt, humus, absorbed Ca + Mg, absorbed Ca, absorbed Mg, P₂O₅, K₂O, NO₃-N, and SO₄ (Kendall’s τ = −0.3178, −0.3197, −0.5728, −0.5077, −0.5103, −0.3377, −0.3193, −0.4702, −0.2985; *p* = 0.0452, 0.0318, 0.0002, 0.0011, 0.0019, 0.0232, 0.0366, 0.0016, 0.0456). Acidobacteria and pH were positively correlated (Kendall’s τ = 0.3530; *p* = 0.0197) but negatively correlated with electrical conductivity, total dissolved solids, and salt (Kendall’s τ = −0.3986, −0.3912, −0.4098; *p* = 0.0071, 0.0082, 0.0098). Abundance of Bacteroidetes were negatively correlated with electrical conductivity, total dissolved solids, and salt (Kendall’s τ = −0.4117, −0.4044, −0.3550; *p* = 0.0050, 0.0058, 0.0240). Abundance of Armatimonadetes were negatively correlated with salt (Kendall’s τ = −0.3912; *p* = 0.0233). Rokubacteria is positively correlated with pH (Kendall’s τ = 0.3471; *p* = 0.0445).

### Fungal identification (ITS)

**Fig. 7:**
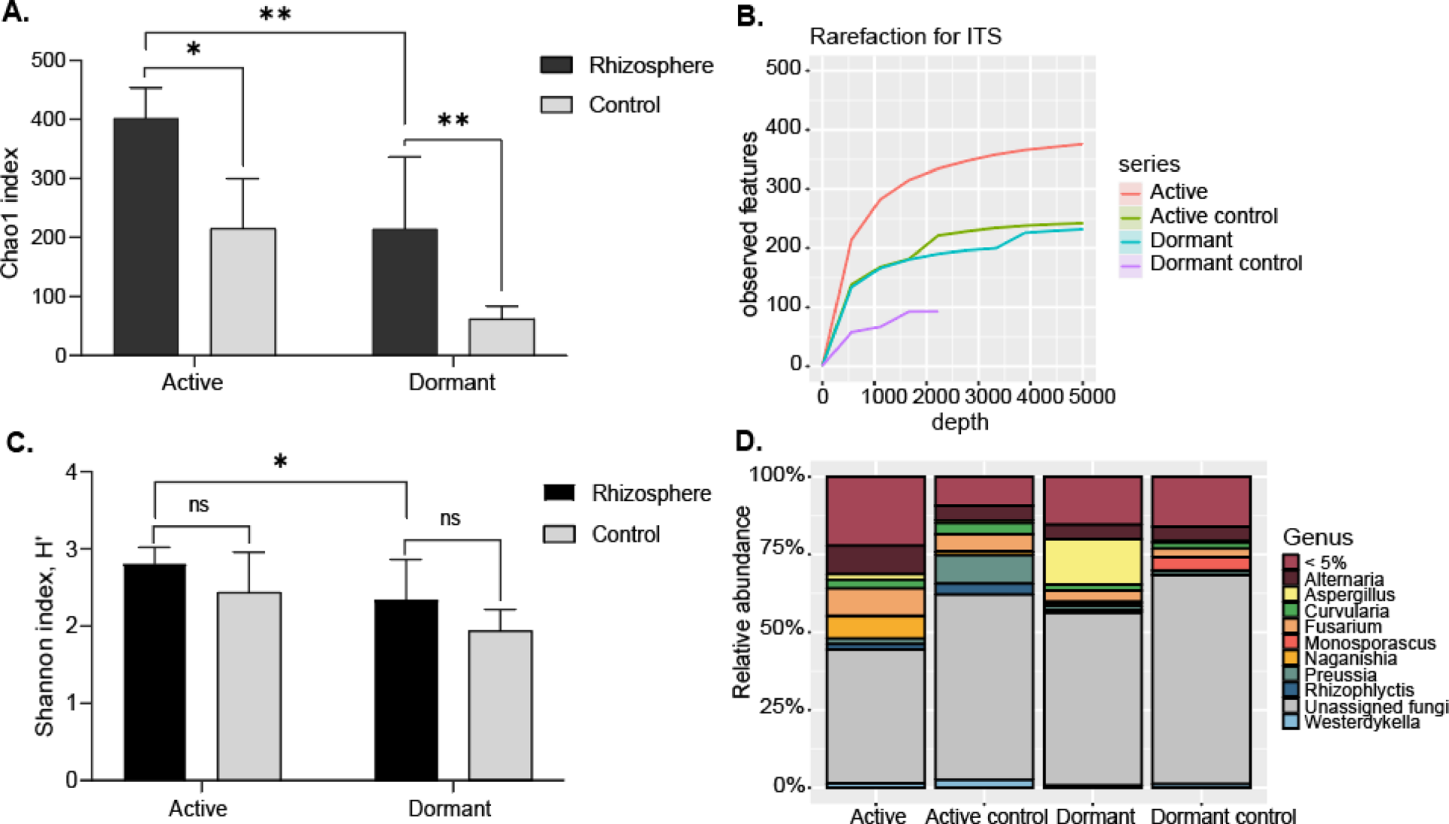
(**A**) Chao index for fungal diversity in the rhizosphere of *Potaninia mongolica* during active and dormant states, compared to their respective controls. The x-axis represents plant states (active, dormant) and their respective controls, while the y-axis shows the Chao index values. Each bar represents the mean Chao index across replicates, with error bars indicating standard deviations. (**B**) Rarefaction curve analysis showing the depth of ITS sequencing of around root soil of active and dormant *Potaninia mongolica*. The x-axis represents the observed features, and the y-axis represents the the sequencing depth. (**C**). Shannon index for fungal diversity in the rhizosphere of *P. mongolica* during active and dormant states, compared to their respective controls. The x-axis shows plant states, and the y-axis represents Shannon index values. Error bars indicate standard deviations. (**D**). Relative abundance of fungi at the genus level. The x-axis represents fungal genus, and the y-axis indicates the relative abundance as a percentage of the total fungal community.

Community richness and diversity of the microbial ecosystem were examined via alpha diversity analysis. Comparing the fungal diversity in the soil around the roots of active and dormant plants revealed that the active state had significantly higher fungal diversity than the dormant state, and the control associated with active plants also had significantly higher diversity than the dormant control (Fig. 7a). The rarefaction curves imply that the sample sizes and depths were sufficient to observe community richness (Fig. 7b). The Shannon index indicated no significant difference in fungal diversity between the rhizosphere of active plants and their respective controls, while the difference between dormant plants and their controls was also not statistically significant. However, fungal diversity differed significantly between the rhizospheres of dormant and active plants (Fig. 7c). The soil around active plant roots was dominated by Ascomycota, followed by Basidiomycota (Fig. 7d) (Supplementary data 2).

The fungal communities of active and active control plants were 75.6 percent similar, while the fungal communities of dormant and dormant control plants were 57.7 percent similar. The index of similarity between active and dormant plants is 83.4 percent. The Bray–Curtis index was 30.8% between active and dormant plant rhizospheres, 36.2% between active plants and their controls, and 7.1% between dormant plants and their respective controls (Table 3).

**Table 3:** Jaccard index of similarity between the active and dormant phases in fungal community.

	**Jaccard index**	**Bray–Curtis index**	**p-value (t-test)**
active vs active control	0.756	0.362	0.0146
active vs dormant	0.834	0.308	0.0028
active vs dormant control	0.411	0.031	0.0001
active control vs dormant	0.82	0.289	0.9936
active control vs dormant control	0.639	0.095	0.0325
dormant vs dormant control	0.577	0.071	0.009

PERMANOVA analysis indicated statistically significant differences in the rhizospheric fungal abundance between the active and dormant plants. There was no statistical difference in rhizospheric fungi abundance between active control and dormant control. In addition, the results below indicate that there is a statistical difference between the dormant and dormant control (Table 4).

**Table 4:** Permutational multivariate analysis of variance (PERMANOVA) table comparing rhizospheric fungal communities in active and dormant plant with their respectively control samples.

Comparisons	df	SS	F.Model	R2	p-value	p-adjusted	sig
*active vs active control*	1	0.67	3.76	0.27	0.004	0.012	
*active vs dormant*	1	1.07	5.41	0.28	0.002	0.01	*
*active vs dormant control*	1	1.69	10.21	0.51	0.003	0.012	
*active control vs dormant*	1	0.50	1.88	0.16	0.019	0.038	
*active control vs dormant control*	1	0.84	3.27	0.35	0.032	0.038	
*dormant vs dormant control*	1	1.10	4.36	0.3	0.001	0.006	*

IndVal identified fifteen species for the active plant soil around the root, including *Bartalinia robillardoides, Filobasidium globisporum* and *Bipolaris melinidis*. In contrast, the IndVal found two indicator species for the active control soil, which included *Sporormiella minimoides* and *Cystobasidium slooffiae*. For dormant plant root soil, the best indicators were *Rhizopus microsporus* and *Monosporascus cannonballus.* IndVal did not find a taxon specific to dormant control. All indicator species as well as their respective indicator values and *p*-values (*p* < 0.05), are *p*resented in Table 5.

**Table 5:** List of indicator species with their IndVal percentage and *p*-value.

States	Species	IndVal	*p-*value
Active	*Bartalinia robillardoides*	0.994	0.001***
	*Filobasidium globisporum*	0.973	0.001***
	*Bipolaris melinidis*	0.935	0.001***
	*Didymella urticicola*	0.921	0.001***
	*Aureobasidium pullulans*	0.904	0.003***
	*Naganishia friedmannii*	0.901	0.005***
	*Aspergillus terreus*	0.89	0.002***
	*Zopfiella latipes*	0.888	0.002***
	*Ceriporia xylostromatoides*	0.855	0.005***
	*Schizothecium inaequale*	0.791	0.01***
	*Nothophoma variabilis*	0.735	0.022*
	*Laetisaria arvalis*	0.72	0.049*
	*Neocamarosporium salsolae*	0.707	0.028*
	*Xenomyrothecium tongaense*	0.707	0.036*
	*Rutstroemia maritima*	0.707	0.034*
Active control	*Sporormiella minimoides*	0.709	0.024*
	*Cystobasidium slooffiae*	0.707	0.038*
Dormant	*Rhizopus microsporus*	0.919	0.008**
	*Monosporascus cannonballus*	0.791	0.014*
Dormant control	-	-	-

**Fig. 8:**
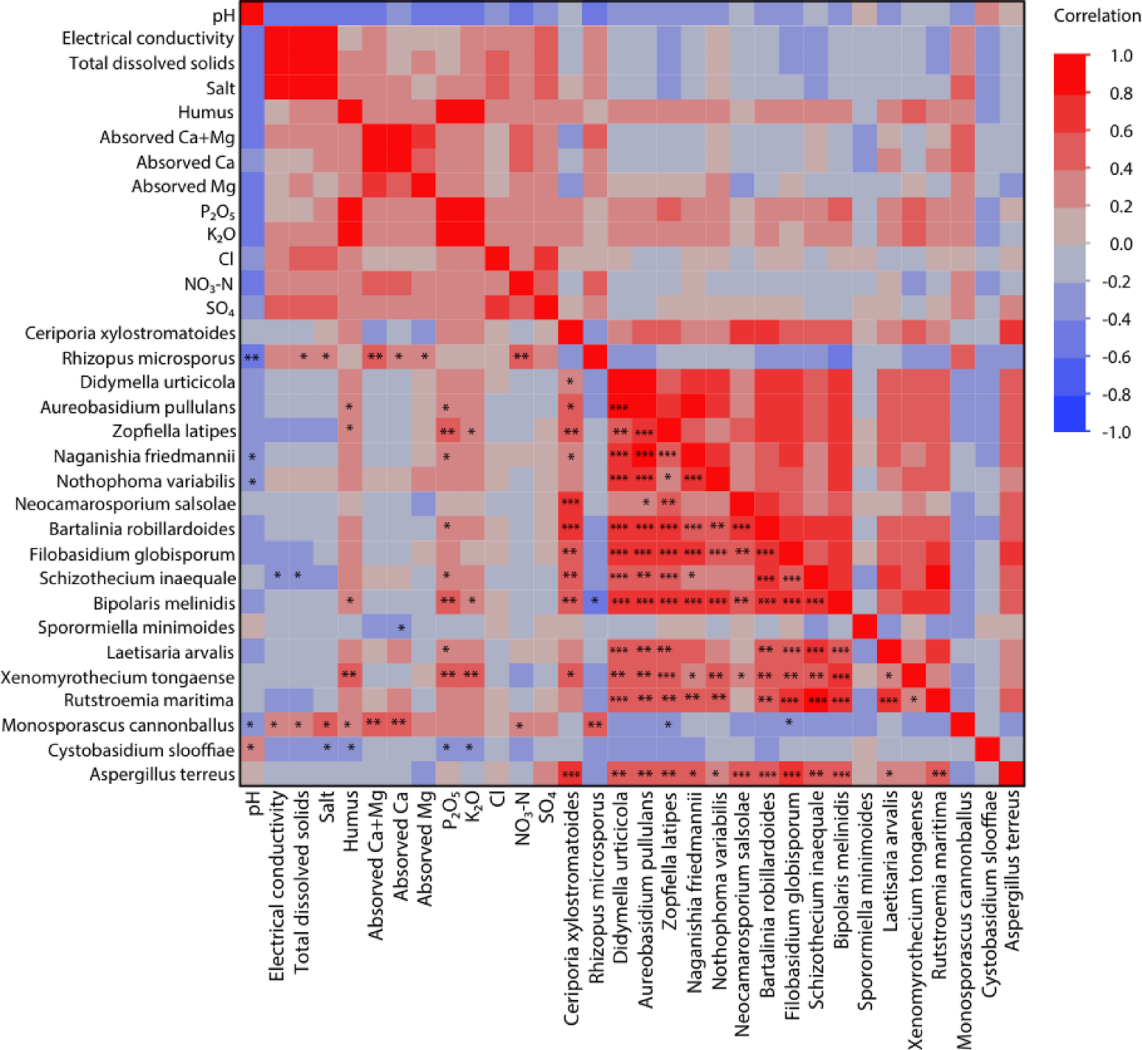
Heatmap of Kendall’s correlation coefficient, presenting associations of indicator fungal abundance with each other and with soil chemical. The heatmap illustrates the strength and direction of correlations, where colors represent the magnitude of Kendall’s coefficient, with warmer colors indicating positive correlations and cooler colors indicating negative correlations.

According to the results of the correlation between the distribution of indicator fungal species and soil chemical properties, *Bartalinia robillardoides*, ranked at the top of the indicator species of the active plant, has a positive correlation with the distribution of *Zopfiella latipes*, *Aureobasidium pullulans*, *Didymella urticicola*, *Ceriporia xylostromatoides*, *Neocamarosporium salsolae*, *Naganishia friedmannii*, *Nothophoma variabilis* and P₂O₅ (Kendall’s τ = 0.7018; 0.6522; 0.6297; 0.6184; 0.6102; 0.5884; 0.462; 0.3749. *p* = < 0.0001; 0.0002; 0.0003; 0.0005; 0.0011; 0.0007; 0.0114; 0.0201) (Fig. 8). Among other chemical factors, humus, K₂O, SO₄^2^ ¯ and Cl ¯ indicate a tendency to have a positive correlation with the distribution of *Bartalinia robillardoides*. The main indicator species of dormant plant, *Rhizopus microspores*, has a positive correlation with NO₃-N, absorbed Ca + Mg, absorbed Ca, salt, absorbed Mg, and total disolved solids (Kendall’s τ = 0.4468; 0.4441; 0.3858; 0.3599; 0.3398; 0.3084; *p* = 0.0058; 0.0078; 0.0229; 0.0369; 0.0567; 0.0548).

## Discussion

While both fungi and bacteria contribute significantly to nutrient cycling in arid soils, their roles differ functionally^29^. Many of the enzymes originate from bacteria and fungi and are essential for maintaining ecosystem functions. Fungi are natural decomposers and are known for their ability to secrete a wide array of extracellular enzymes involved in the breakdown of complex organic matter^30^. Fungal cells exhibit both intracellular and extracellular enzymatic activity, with many enzymes particularly hydrolases and oxidoreductases playing critical roles in substrate bioconversion and environmental resilience^31,32^. Among these, laccases and peroxidases are especially important for degrading recalcitrant compounds and detoxifying harmful substances in both natural and industrial contexts^33,34^. Fungi also protect the plant by creating a protective sheath around the plant root during moisture stress and provides moisture to the plant through the fungal hyphal network^35^. In contrast, bacteria are often dominant in processes related to nitrogen cycling, such as nitrification and denitrification, through enzymes like urease and nitrate reductase^36,37^. The results of our study showed that the fungal diversity of the active and dormant communities differed from their respective control, while the bacterial diversity did not. In our study, Ascomycota and Basidiomycota were the dominant fungal phyla, while Proteobacteria and Actinobacteria were most abundant among bacteria consistent with microbial community compositions reported by Xie et al*.*^38^. Furthermore, we found that fungal diversity varied more significantly with plant physiological state, while bacterial communities showed no difference between active and dormant states, aligning with the findings of Lu et al.^39^ who observed similar microbial responses in arid ecosystems. Our observations also support the conclusions of Li et al.^40^ who inferred that under drought conditions, soil microorganisms may adopt nitrogen utilization strategies by increasing ammonium nitrogen availability, thereby enriching the rhizosphere to support plant function. In line with this, our data suggest that nitrogen supplying fungi may play a protective role in maintaining dormant *P. mongolica* plants during physiologically inactive periods, potentially preventing desiccation-induced mortality through continued nitrogen provisioning.

Microorganisms compete with each other, but in order to co-exist, fungi respond to ecological succession using a variety of strategies, such as compensating for each other’s metabolic deficits when they cannot meet the needs of the host organism. Soil nutrients are key factors influencing fungal abundance and community strategy^41^. For instance, in an experiment examining the effect of nutrients on red soil, it was found that soil chemical parameters, especially pH, have a significant effect on fungal community composition^42^. The chloride salt also enhances plant resistance to pathogens and biotic stresses^43^. The significantly higher electrical conductivity, total disolved solids and salt levels in dormant plant soil are indicators of each other^44^, and Gobi soils are characterized by high salt content, and Gobi plants are highly tolerant to salt^45^. The results of the study indicate that the higher NO₃-N levels in dormant plants are related to their rhizospheric microbiome. Although nitrogen dynamics were analyzed, we acknowledge that microbial processes such as nitrification and denitrification may influence nitrogen availability and loss. These were not directly measured in this study and may act as confounding factors. Future work should consider incorporating microbial assays to better understand their role in nitrogen cycling. The abundance of the second indicator species, *Monosporascus cannonballus*, is positively correlated with the abundance of *Rhizopus microsporus* species and chemical parameters led by Absorbed Ca + Mg. However, the abundance of indicator fungal species in the rhizosphere of active plants had a significant positive correlation with each other, rather than with soil chemicals. From this, it can be seen that the abundance of rhizospheric fungal species in the biologically active plants is positively correlated with each other, while the abundance of rhizospheric fungi in the biologically inactive state is directly related to the soil chemicals, which have an important effect on plant growth. *Rhizopus microsporus* was detected in higher abundance, suggesting a potential ecological association with stress-adapted rhizospheres. However, further experimental validation such as controlled inoculation assays is needed to confirm any causal role in drought tolerance.

The number of indicator species in the active state was greater than that of the dormant plants, which may indicate that living near the biologically active plants presents a favourable environment for fungi. Furthermore, during dormancy, fungi can provide metabolic support and protect plants from diseases. Therefore, the individual characteristics of microbiomes that interact with plants are certainly of interest. For instance, *Rhizopus microsporus*, an indicator fungi species of dormant plants, is a plant growth-promoting fungus^46^. The second fungal indicator species of dormant plant state, *Monosporascus cannonballus*, is highly tolerant of dry, high temperature, and saline environments and can survive at a pH of 9^47^.

An indicator fungal species *Bartalinia robillardoides* in the active plant is commonly found in freshwater and synthesizes compounds used in antitumor drugs^48^. The second indicator, *Filobasidium globisporum* was also found in rhizosphere of *Atractylodes lancea*, a medicinal plant^49^. Third indicator *Aureobasidium pullulans* produces melanin and pullulan polymers with physicochemical properties that can be used in a variety of applications in the food, pharmaceutical, and cosmetic industries^50,51^. This family is able to survive heavy metals by secreting melanin^50,52^. It was also found that *A. pullulans* supported the growth of beans and soybeans Francesco et al.^53^. The next indicator species, *Naganishia friedmannii*, has a high tolerance to extremely low temperature, and high solar irradiation, acidic soils and low moisture conditions^54,55^. The next indicator species, *Aspergillus terreus*, is commonly found in soil and decaying organic matter and attracts the attention of researchers because it produces secondary metabolites such as mycotoxins and antibiotics^56^. Following species, *Zopfiella latipes*, shows activity against gram-positive bacteria and fungi, and antimicrobial compounds have been extracted from it^57^. The consecutive indicator species, *Ceriporia xylostromatoides*, plays an important role in the decomposition process of woody plants^58^. The subsequent indicator, *Schizothecium inaequale*, was noted in the early stages of saw decomposition and described as a coprophilous and endophytic fungus^59^. The next in line, *Nothophoma variabilis* species, is abundant in woody plants^60,61^. The following indicator species, *Laetisaria arvalis*, has extraordinary cellulose-deconstructing capacity of the basidiomycete^62^. The next indicator species, *Neocamarosporium salsolae*, is halotolerant and occurs mostly in saline soils^63^. The following indicator species, *Xenomyrothecium tongaense*, shows antibacterial activity^64^. Although there is less information about the next indicator species *Rutstroemia maritima*, some species belonging to this genus have been reported to affect premature senescence and sterility of *Bromus tectorum*, but not on fertilization and growth^65^.

In active control plant rhizosphere, *Sporormiella minimoides* produces compounds with antifungal activity^66^. The yeast isolated from the next indicator species, *Cystobasidium slooffiae*, can simultaneously produce bio-hydrogen and bio-electricity from xylose, which would diversify the toolbox of biomass energy^67^.

The active plant rhizospheric bacterial indicator species *Lechevalieria aerocolonigenes* belongs to the Pseudonocardiaceae family, which is the producer of the indolocarbazole antitumor antibiotic rebeccamycin^68^. The next indicator species belongs to the Kineococcus genus, which is resistant to saline soils and environments with high temperature fluctuations, and this genus is also isolated from the roots of medicinal plants^69^. The alanine racemase enzyme isolated from the Chitinophagaceae bacterium, which belongs to the next indicator species, is a key target for the development of antimicrobial drugs or inhibitor design^70^. The next indicator species, *Belnapia rosea*, tolerates 1% (w/v) NaCl and exhibits good growth at temperatures ranging from 28 to 32 °C and a pH of 7.0–7.5^71^. Tolerates 1% (w/v) NaCl. Growth occurs at pH 5.0–8.0 and 20–37 °C. Good growth occurs at 28–32 °C and pH 7.0–7.5, respectively. The optimum growth temperature for the genus of the next indicator species, *Microvirga subterranea*, is 41 °C, with a growth temperature range between 25 and 45 °C. Additionally, its cells are susceptible to antibiotics that target members of the Bacteria domain^72^. The genus of the next indicator species, Microbacterium yannicii PS01, is widely distributed across various regions. It is resistant to heavy metals and, like other Microbacterium strains, produces siderophores, ACC deaminase, auxins (IAA), and can solubilize phosphate^73^.

The genus Bacillus, an active control plant rhizospheric bacterial species, is a focus of researchers due to its physiological adaptation as an alkaliphile^74^. *Bacillus foraminis* demonstrates strong resistance to potentially toxic elements and was successfully utilized for the first time in the bioleaching of AMOLED mobile displays^75^. The next indicator species, *Blastococcus saxobsidens*, exhibited a greater resitence to Cr^2^⁺ and Cu^2^⁺^76^.

Dormant plant indicator rhizospheric bacterial species belongs to the Pseudomonadaceae family, which plays an important role in suppressing and protecting against root rot disease^77^. Dormant control plant indicator bacterial species, *Ramlibacter tataouinensis TTB310i*, has developed a complex network of two-component systems that may respond to light and possibly employ a rudimentary circadian hourglass. This allows the bacteria to anticipate water availability during the dew period in the middle or end of desert winter nights, thereby aligning its growth cycle with times of cyclic water availability (De Luca et al., ^85^). The next indicator belongs to the Nitrospira genus. The discovery and cultivation of a fully nitrifying bacterium from the genus Nitrospira, a globally distributed group of nitrite oxidizers, revealed that its genome encodes pathways for both ammonia and nitrite oxidation. These pathways are simultaneously activated during growth, as ammonia is oxidized to nitrate^78^. The next indicator bacterium contains Clustered Regularly Interspaced Short Palindromic Repeats (CRISPR), which functions as an adaptive immune system against mobile genetic elements in many prokaryotes, as well as Trehalose, which has significant applications in the food industry and pharmaceutical manufacturing^79^^,^^80^.

Nutrients play a crucial role in the biomass and pasture quality of Gobi plants, but fertilizing large areas with fertilizers is unrealistic^81^. Moreover, depending on the type of fertilizer, the soil’s acidity can increase, which presents a risk of damaging seeds during germination^82^. Additionally, it has become increasingly difficult to cultivate rare and endangered species in the Gobi Desert, which poses challenges for restoration and conservation efforts. Therefore, there is a continued need for experimental research to develop organic fertilizers using rhizospheric fungi or bacteria, which can positively affect plant nutrient uptake, and to isolate beneficial rhizospheric fungi or bacteria for research.

The findings suggest that the rhizospheric fungal communities associated with active *Potaninia mongolica* are more diverse in terms of indicator species than those associated with dormant plants. This higher diversity in active plants may reflect a more dynamic and metabolically active rhizosphere, where the plant is engaging in processes such as nutrient uptake, growth, and interaction with soil microbiomes. Active plants typically require a broader range of microbial species to support their physiological needs, such as nutrient acquisition, growth, and protection against pathogens, which could explain the greater number of fungal indicator species.

The fungal species associated with active plants appear to be independent of soil chemical properties, suggesting that these microbial communities may be influenced by factors other than the soil chemical composition, such as plant-specific root exudates or microbial interactions. This could imply that active plants maintain a functional rhizosphere through mechanisms other than soil nutrient availability, such as chemical signaling or competition among microbes.

In contrast, the fungi associated with dormant plants show a positive correlation with soil chemicals, particularly nutrients. During dormancy, the plant is in a low metabolic state, which may cause it to rely more on soil nutrients for survival. The positive relationship between dormant plant fungi and soil chemicals indicates that these fungi may play an important role in nutrient cycling or bioavailability in the rhizosphere. They could help in the decomposition of organic matter or facilitate nutrient uptake, which becomes critical for the dormant plant as it conserves energy and water during harsh environmental conditions.

Overall, the findings suggest that the microbial communities in the rhizospheres of active and dormant plants may function differently in relation to their environment. Active plants foster a more diverse microbial community that is less dependent on soil chemical composition, whereas dormant plants rely more on the soil chemical properties, and the fungi associated with them may be more specialized for nutrient cycling and maintaining soil health during dormancy. Led by indicator species such as *Rhizopus microsporus*, *Monosporascus cannonballus*, and members of the *Pseudomonadaceae* family, the identified microbial taxa may serve as promising candidates for bioinoculant development. These organisms could support drought-adapted plant growth.

These observations have important implications for understanding how plants manage their microbial partners under different environmental conditions and how microbial communities contribute to plant health and survival. The results also highlight the potential for manipulating soil microbial communities to support plant health in both active and dormant states. Manipulating soil microbiomes presents an exciting avenue for enhancing plant health, stress tolerance, and nutrient cycling. However, translating such approaches to field-scale applications involves significant challenges. The complexity and resilience of native microbial communities, coupled with environmental variability, can limit the persistence and effectiveness of introduced or engineered microbiomes. Moreover, practical implementation at scale requires consideration of ecological compatibility, delivery methods, and long-term monitoring. While our findings support the potential value of microbiome-based interventions, further research is needed to develop robust, context-specific strategies for their application under real-world conditions. Interpretation of our findings should consider key limitations. Sampling during a single season may not capture temporal shifts in microbial communities and biogeochemical processes. Additionally, unaccounted spatial heterogeneity may influence microbial composition due to localized environmental variation. Future studies with multi-seasonal and spatially stratified designs will improve ecological resolution.

## Supplementary Information

Supplementary Information 1.

Supplementary Information 2.

## Data Availability

The raw data for 16S and ITS high-throughput amplicon sequencing are available at NCBI SRA under the accession number PRJNA1208213. Associated metadata in this study are provided in the Supplementary Data file.

## Code availability

The core scripts used for 16S rRNA and ITS taxonomic analyses, together with supporting routines for statistical evaluation, have been archived in Figshare and are publicly accessible at 10.6084/m9.figshare.30091735.v1.
